# DNA 5mC and RNA m^6^A Collaborate to Upregulate Phosphoenolpyruvate Carboxykinase 2 for Kupffer Cell Activation

**DOI:** 10.3390/ijms25189894

**Published:** 2024-09-13

**Authors:** Yulan Zhao, Wenbo Yuan, Yue Feng, Ruqian Zhao

**Affiliations:** 1 MOE Joint International Research Laboratory of Animal Health & Food Safety, Nanjing Agricultural University, Nanjing 210095, China; 2 Key Laboratory of Animal Physiology & Biochemistry, College of Veterinary Medicine, Nanjing Agricultural University, Nanjing 210095, China

**Keywords:** KCs, 5mC, m^6^A, Inflammation, PCK2

## Abstract

Both DNA 5-methylcytosine (5mC) and RNA N6-methyladenosine (m^6^A) modifications are reported to participate in cellular stress responses including inflammation. Phosphoenolpyruvate carboxykinase 2 (PCK2) is upregulated in Kupffer cells (KCs) to facilitate the proinflammatory phosphorylation signaling cascades upon LPS stimulation, yet the role of 5mC and m^6^A in PCK2 upregulation remain elusive. Here, we report that the significantly augmented PCK2 mRNA and protein levels are associated with global 5mC demethylation coupled with m^6^A hypermethylation in LPS-activated KCs. The suppression of 5mC demethylation or m^6^A hypermethylation significantly alleviates the upregulation of PCK2 and proinflammatory cytokines in LPS-challenged KCs. Further reciprocal tests indicate 5mC demethylation is upstream of m^6^A hypermethylation. Specifically, CpG islands in the promoters of PCK2 and RNA methyltransferase (METTL3 and METTL14) genes are demethylated, while the 3′UTR of PCK2 mRNA is m6A hypermethylated, in LPS-stimulated KCs. These modifications contribute to the transactivation of the PCK2 gene as well as increased PCK2 mRNA stability and protein production via a m^6^A-mediated mechanism with IGF2BP1 as the reader protein. These results indicate that DNA 5mC and RNA m6A collaborate to upregulate PCK2 expression, respectively, at the transcriptional and post-transcriptional levels during KC activation.

## 1. Introduction

Hepatic inflammation stands as a pivotal juncture in the pathological trajectory of chronic liver diseases, encompassing fibrosis, cirrhosis, and cancer [1,2]. This inflammatory cascade is ignited by the activation of Kupffer cells (KCs), the resident macrophages of the liver [3,4]. Such activation triggers metabolic adaptations akin to the Warburg effect, wherein metabolic reprogramming redirects energy production towards supporting the immune response [5]. Specifically, gluconeogenesis and glycolysis collaborate as primary energy-generating pathways, fueling the inflammatory response and the activation of immune cells. Emerging evidence underscores the intricate interplay between metabolic enzymes and the inflammatory machinery of macrophages. For instance, the dimeric pyruvate kinase M2, the glycolytic rate-limiting enzyme, can translocate into the nucleus where it interacts with Hif1-α to promote IL-1β production in LPS-activated macrophages [6]. Similarly, our previous findings revealed that the gluconeogenesis enzyme, phosphoenolpyruvate carboxykinase 2 (PCK2), is upregulated in KCs upon LPS exposure. This upregulation facilitates the protein phosphorylation of key signaling molecules such as NF-κB and AKT/MAPK, thereby potentiating the inflammatory response [7]. Despite these insights, the molecular mechanisms that underlie PCK2 upregulation in LPS-activated KCs remain shrouded in mystery.

Both DNA 5-methylcytosine (5mC) and RNA N6-methyladenosine (m^6^A) modifications are reported to play a role in the epigenetic regulation of gene expression during the pathogenesis of various diseases, including liver fibrosis [8] and cancer [9]. Increasing evidence has revealed a close association between epigenetic regulation and inflammatory response. For instance, DNA demethylase ten-eleven translocation 2 (TET2) recruits histone deacetylase HDAC2 to repress IL-6 transcription via histone deacetylation during the resolution phase of inflammation in innate myeloid cells, including dendritic cells and macrophages [10]. In LPS-activated KCs, NF-κB p65 transactivates the expression of m^6^A methyltransferases METTL3 and METTL14, which in turn increases m^6^A modification in the 5′UTR of TGF-β mRNA to promote m^6^A-mediated TGF-β translation in a cap-independent manner [11]. Although both 5mC and m6A have been implicated in immune activation processes, the extent to which these epigenetic modifications contribute to the upregulation of PCK2 specifically in activated Kupffer cells (KCs), and the mechanisms involved, remain largely unexplored and worthy of further investigation.

In this study, we show that PCK2 upregulation in LPS-activated KCs is associated with global DNA hypomethylation and RNA m^6^A hypermethylation. Locus-/site-specific analyses reveal decreased 5mC modification on the CpG island of the PCK2 gene promoter together with increased m^6^A modification on the 3′UTR of PCK2 mRNA in activated KCs. Further functional validation assays confirm the role of 5mC and m^6^A in the upregulation of the PCK2 gene, respectively, at the transcriptional and post-transcriptional levels. IGF2BP1 is identified as an m^6^A reader protein to enhance PCK2 mRNA stability. Finally, we discover a collaborative mechanism between 5mC and m^6^A modification in LPS-activated KCs.

## 2. Results

### 2.1. Metabolic Reprogramming in LPS-Activated KCs Is Associated with PCK2 Upregulation

**Figure 1:**
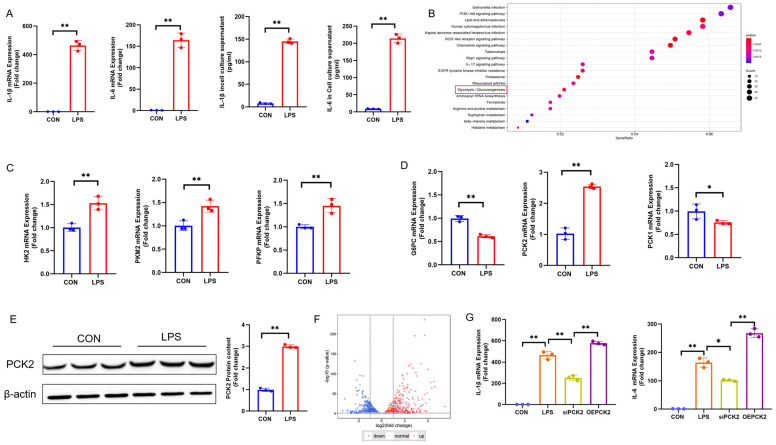
Gluconeogenesis/glycolytic pathway variation in LPS-activated KCs is associated with PCK2 upregulation. (**A**). IL-6 and IL-1β mRNA expression. IL-6 and IL-1β levels in culture media. (n = 3). (**B**) RNA-sequencing Kyoto Encyclopedia of Genes and Genomes (KEGG) enrichment analysis of transcripts expression. (**C**) HK2, PFKP, and PKM2 mRNA expression (n = 3). (**D**) G6PC, PCK1, and PCK2 mRNA expression. (**E**) PCK2 protein expression (n = 3). (**F**) RNA-sequencing show that compared with PCK2 were significantly upregulated in LPS group; (**G**) Expression of IL-1β and IL-6 after inhibition of PCK2 and overexpression of PCK2. Values are means ± SE, * *p* < 0.05 and ** *p* < 0.01.

LPS led to elevated IL-6 and IL-1β levels in culture media and an upregulation of their respective mRNAs in the cells (Figure 1A). The KEGG pathway enrichment of published databases showed that KC activation was associated with significant changes in the gluconeogenesis/glycolytic pathway by LPS (Figure 1B). In LPS-activated KCs, the glycolytic enzymes hexokinase 2 (HK2), phosphofructokinase P (PFKP), and pyruvate kinase M2 isoform (PKM2) were significantly increased at the mRNA level (Figure 1C), suggesting metabolic reprogramming during KC activation. The gluconeogenesis enzymes phosphoenolpyruvate carboxykinase 1 (PCK1) and glucose-6-phosphatase(G6PC) were significantly downregulated (Figure 1D), whereas PCK2 was significantly upregulated, at both the mRNA and protein (Figure 1E) levels. Volcano mapping of differentially expressed genes in the publicly available database showed that PCK2 was also significantly upregulated (Figure 1F). We knocked down PCK2-reduced LPS-induced IL-6 and IL-1β mRNA, and overexpressed increased LPS-induced IL-6 and IL-1β mRNA (Figure 1G).

### 2.2. PCK2 Upregulation in LPS-Activated KCs Is Associated with Global DNA 5mC Demethylation and RNA m^6^A Hypermethylation

**Figure 2:**
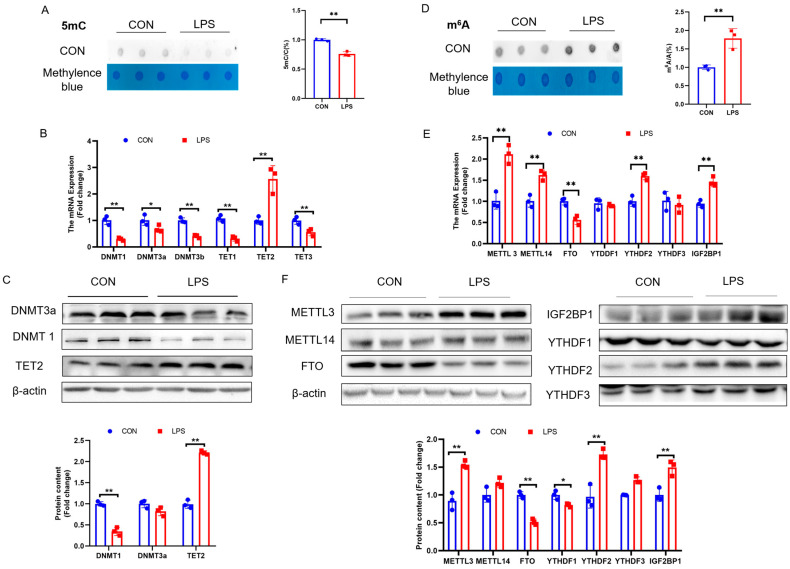
(**A**) Total DNA 5mC modification (n = 3). (**B**) DNMT1, DNMT3A, DNMT3b, TET1, TET2, and TET3 mRNA expression (n = 3). (**C**) DNMT1, DNMT3A, and TET2 protein expression (n = 3). (**D**) Total RNA m^6^A modification (n = 3). (**E**) METTL3, METTL14, FTO, YTHDF1, YTHDF2, YTHDF3, and IGF2BP1 mRNA expression (n = 3). (**F**) METTL3, METTL14, FTO, YTHDF1, YTHDF2, YTHDF3, and IGF2BP1 protein expression (n = 3). Values are means ± SE, * *p* < 0.05 and ** *p* < 0.01.

KC activation is associated with significant global 5mC demethylation (Figure 2A), which was supported by the significant downregulation of DNA methyltransferases (DNMT1, DNMT3a, and DNMT3b), together with the significant upregulation of DNA demethylase TET2 at both the mRNA (Figure 2B) and protein (Figure 2C) levels. Simultaneously, significant global m^6^A hypermethylation was detected in LPS-activated KCs (Figure 2D), which was accompanied by METTL3 and METTL14 upregulation and FTO downregulation, at both the mRNA (Figure 2E) and protein levels (Figure 2F). 

### 2.3. Suppression of 5mC Demethylation and m^6^A Hypermethylation Rectifies LPS-Induced PCK2 Upregulation

**Figure 3:**
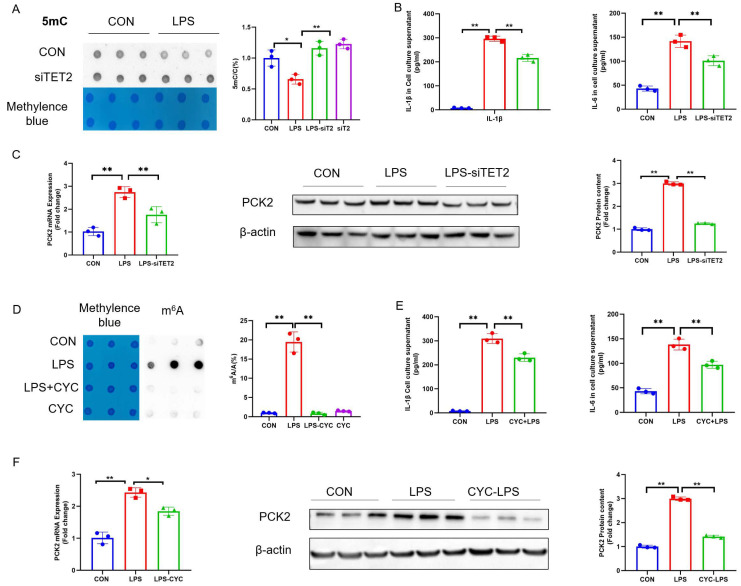
Suppression of 5mC demethylation and m6A hypermethylation rectifies LPS-induced PCK2 upregulation. (**A**) Total DNA 5mC modification after TET2 knockdown (n = 3). (**B**) IL-6 and IL-1β levels in culture media after TET2 knockdown (n = 3). (**C**) mRNA and protein levels of PCK2 (n = 3). (**D**) Total RNA m^6^A modification after treatment with CYC (n = 3). (**E**) IL-6 and IL-1β levels in culture media after treatment with CYC (n = 3). (**F**) mRNA and protein levels of PCK2 after treatment with CYC. Values are means ± SE, * *p* < 0.05 and ** *p* < 0.01.

To understand whether 5mC is involved in the upregulation of PCK2 in LPS-activated KCs, we transfected KCs with siTET2 to prohibit 5mC demethylation, the knockdown effect of TET2 is shown in Figure S1A. The knockdown of TET2 reversed LPS-induced 5mC hypomethylation (Figure 3A) and reduced the LPS-induced increase in IL-1β and IL-6 concentrations in culture media (Figure 3B). In addition, TET2 knockdown reduced LPS-induced PCK2 upregulation at both the mRNA and protein levels (Figure 3C). 

Similarly, to explore whether m^6^A is involved in the upregulation of PCK2 in LPS-activated KCs, KCs were treated with m^6^A inhibitor cycloleucine (CYC) to prevent m^6^A hypermethylation. CYC treatment protected KCs from an LPS-induced increase in m^6^A modification (Figure 3D) and reduced IL-1β and IL-6 levels in culture media (Figure 3E). In addition, CYC rescued LPS-induced PCK2 elevation at both the mRNA and protein levels (Figure 3F). 

### 2.4. CpG Island of PCK2 Promoter Is 5mC-Demethylated and 3′UTR of PCK2 mRNA Is m^6^A-Hypermethylated in LPS-Activated KCs

**Figure 4:**
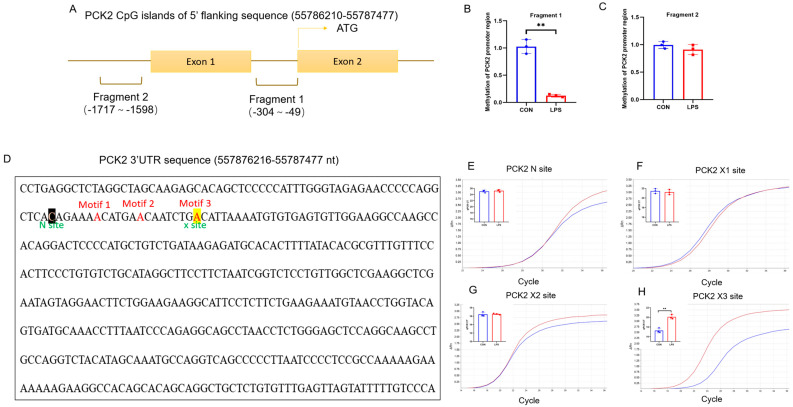
CpG island of PCK2 promoter is 5mC-demethylated and 3′UTR of PCK2 mRNA is m6A-hypermethylated in LPS-activated KCs. (**A**) Schematic diagram of CpG islands on the promoter of PCK2 gene. (**B**) 5mC is reduced in fragment 1 (−304~−49) of PCK2 gene promoter region (n = 3). (**C**) 5mC has not change in fragment 1 (−1717~−1590) of PCK2 gene promoter region (n = 3). (**D**) The m^6^A site was predicted for the PCK2 3′UTR. The RRACU-compliant motif was named motif 1–3 (X1–X3 site); a non-m6A modification site of this mRNA was selected as the N Site. (**E**–**H**) No significant changes in cycle of threshold (Ct) were determined at N, X1, and X2 sites, while a significant increase in PCK2 3′UTR at the X3 site was observed. Values are means ± SE, ** *p* < 0.01.

Two CpG islands on the 5′-flanking sequences (−5000~0) of the PCK2 gene were predicted using the Methprimer (Figure 4A). Two pairs of primers were designed on sequences (−304~−49 and −1717~−1590) to determine the level of 5mC modification on these two CpG islands by MeDIP-PCR. Among the two fragments analyzed, fragment 1 (−304~−49) in the proximal CpG island of the PCK2 gene promoter was hypomethylated (Figure 4B), while the 5mC level on the fragment 2 (−1717~−1590) was unchanged (Figure 4C).

Three high-potential m^6^A sites on PCK2 3′UTR at +1992, +1998, and +2006 were predicted with SRAMP and referred to as X1, X2, and X3, respectively (Figure 4D). A non-m^6^A modification site of this mRNA was selected as the N Site, which was used as an internal reference in the SELECT assay for the quantification of the m^6^A level on the predicted high-potential modification sites. No significant changes in the cycle of threshold (Ct) were determined at the N (Figure 4E), X1 (Figure 4F), or X2 sites (Figure 4G), while a significant increase in PCK2 3′UTR the X3 site (Figure 4H) was detected in LPS-treated KCs.

### 2.5. 5mC Demethylation and m^6^A Hypermethylation Contribute Collectively to PCK2 Upregulation in LPS-Activated KCs

**Figure 5:**
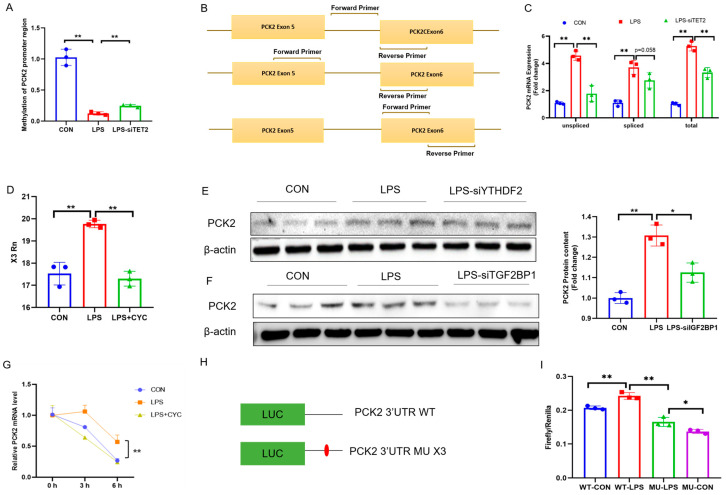
Locus-/site-specific 5mC demethylation and m6A hypermethylation contribute collectively to PCK2 upregulation in LPS-activated KCs. (**A**) 5mC increased in fragment 1 (−304~−49) of PCK2 gene promoter region after TET2 knockdown (n = 3). (**B**) TET2 knockdown reduced the increase in total mRNA in PCK2 induced by LPS (n = 3). (**C**) TET2 knockdown reduced the increase in un-spliced mRNA in PCK2 induced by LPS (n = 3). (**D**) P TET2 knockdown reduced the increase in spliced mRNA in PCK2 induced by LPS (n = 3). (**E**) CYC treatment rectified LPS-induced m6A hypermethylation on X3 site of PCK2 3′UTR (n = 3). (**F**) Knockdown IGF2BP1 mitigated PCK2 upregulation in LPS-activated KCs (n = 3). (**G**) CYC treatment reduced LPS resulting in increased PCK2 mRNA stability (n = 3). (**H**) Schematic representation of synthetic mRNAs containing PCK2 3′UTR and full-length firefly luciferase. (**I**) Synthetic mRNAs were transfected, and luciferase activity was detected in KCs. Values are means ± SE, * *p* < 0.05 and ** *p* < 0.01.

The knockdown of TET2 significantly alleviated LPS-induced 5mC demethylation on the CpG island of the PCK2 gene promoter (Figure 5A). The primers were designed to specifically amplify the total, un-spliced, and spliced PCK2 mRNA (Figure 5B). TET2 knockdown significantly alleviated an LPS-induced increase in all the three species of PCK2 mRNA, while un-spliced PCK2 mRNA showed a more dramatic reversal (Figure 5C), suggesting the role of 5mC modification in the transcriptional activation of PCK2 in LPS-activated KCs.

Concurrently, the LPS-induced m^6^A hypermethylation of PCK2 3′UTR on the X3 site was also rectified by CYC treatment (Figure 5D). To validate the function of potential reader proteins, the two significantly upregulated m^6^A reader proteins, YTHDF2 and IGF2BP1, were knocked down by siRNAs. The knockdown of YTHDF2 failed to restore an LPS-induced increase in PCK2 (Figure 5E and Figure S1B), whereas the knockdown of IGF2BP1 significantly mitigated PCK2 upregulation in LPS-activated KCs (Figure 5F). These results provide the evidence for IGF2BP1/m^6^A-mediated post-transcriptional PCK2 upregulation in LPS-activated KCs. The mRNA stability analysis revealed a significantly prolonged PCK2 mRNA lifetime in LPS-treated KCs, which was restored by CYC treatment (Figure 5G). Finally, a luciferase reporter assay with plasmids carrying a wild-type (WT) or X3 site-mutated (A to T) 3′UTR of PCK2 mRNA (Figure 5H) confirmed the role of this specific m^6^A site in the LPS-induced increase in PCK2 3′UTR luciferase activity (Figure 5I).

### 2.6. METTL3/METTL14 Genes Are Transactivated in LPS-Activated KCs via Promoter 5mC Hypomethylation

**Figure 6:**
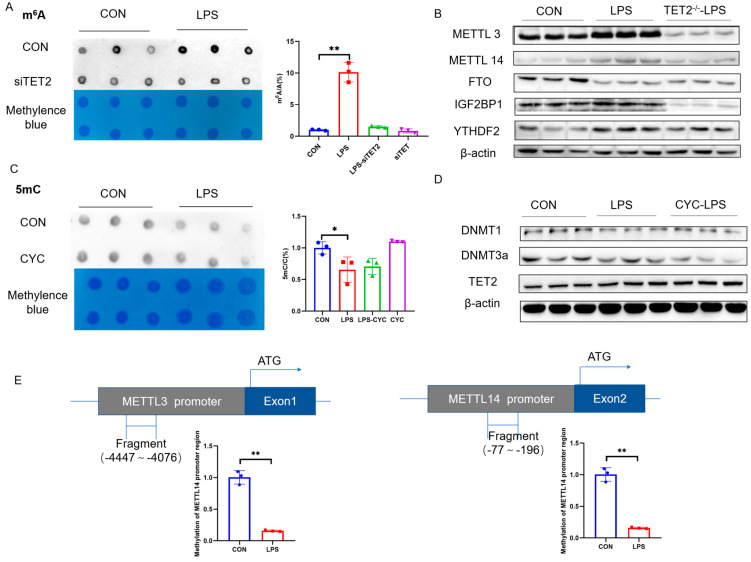
METTL3/METTL14 genes are transactivated in LPS-activated KCs via promoter 5mC hypomethylation. (**A**) Total RNA m^6^A modification after TET2 knockdown (n = 3). (**B**) METTL3, METTL14, FTO, YTHDF1, YTHDF2, YTHDF3, and IGF2BP1 protein expression after TET2 knockdown (n = 3). (**C**) Total DNA 5mC modification after treatment with CYC (n = 3). (**D**) DNMT1, DNMT3A, and TET2 protein expression (n = 3) after treatment with CYC. (**E**) METTL3 and METTL14 mRNA expression after treatment with the 5mc inhibitor 5aza (n = 3). Schematic diagram of CpG islands on the promoter of METTL3 and METTL14 gene, and 5mC is reduced in METTL3 and METTL14 gene promoter region (n = 3). Values are means ± SE, * *p* < 0.05 and ** *p* < 0.01.

The knockdown of TET2, which increases the total 5mC modification, protected KCs from an LPS-induced increase in total m^6^A modification (Figure 6A), which was supported by accordant alterations of m^6^A methyltransferases METTL3 and METTL14 (Figure 6B and Figure S1C). Nevertheless, the reciprocal inhibition of m^6^A with CYC failed to protect KCs from an LPS-induced decrease in 5mC modification (Figure 6C) or corresponding changes in DNA methyltransferases DNMT1/DNMT3a or DNA demethylase TET2mRNA and protein levels (Figure S1D). These results indicate a relationship between 5mC and m^6^A modifications, with 5mC being upstream of m^6^A. The treatment of KCs with 5aza also increased METTL3 and METTL14 mRNA expression (Figure 6D). Indeed, two CpG islands were predicted, respectively, on 5′-flanking sequences of METTL3 (−5000~0) and METTL14 (−5000~0) gene promoters. LPS induced significant demethylation on predicted CpG islands of the METTL3 and METTL14 gene promoters, which was restored by TET2 knockdown (Figure 6E). These findings indicate that LPS induces 5mC demethylation on METTL3/METTL14 gene promoters, which contributes to the transcriptional activation of these genes to produce m^6^A hypermethylation in KCs.

## 3. Discussion

Gluconeogenesis is involved in the development of type 2 diabetes [12] and cancer [13]. PCK1, the key enzyme in gluconeogenesis, was found to promote the development of lipid deposition inflammation and fibrosis in the mouse MAFLD model [14]. Previously, we also reported that the mitochondrial isoform PCK2 is activated in KCs upon LPS stimulation to facilitate the inflammatory response in the liver [7]. Here, we went on to explore the molecular mechanism underlying the upregulation of PCK2 in LPS-activated KCs. We provide the evidence for the participation and collaboration of 5mC demethylation and m^6^A hypermethylation in the regulation of PCK2 expression, respectively, at the transcriptional and post-transcriptional levels.

Several studies suggest a role of 5mC in immune response. For example, intrapulmonary capillary-derived Rspondin3 promotes the conversion of pulmonary interstitial macrophages to an anti-inflammatory phenotype, which is accompanied by metabolic and epigenetic remodeling. In this process, epigenetic remodeling is mainly manifested by TET2-induced DNA hydroxy methylation enhancement [15]. DNA hydroxymethylation is the oxidation of 5mC to 5-hydroxymethylcytosine (5hmC) catalyzed by TET family enzymes, which is the main pathway of 5mC reduction. In our model of KCs activation, 5mC showed demethylation with the concomitant elevation of TET2. Indeed, the decrease in 5mC caused by the demethylase TET2 was also confirmed to be a key factor in the regulation of inflammation by 5mC [16]. For example, CXXC finger protein 5 (CXXC5) recruits TET2 to maintain the hypomethylation of dendritic cell genomic CpG islands and the hypomethylation of IRF7 promoter promotes transcription, thereby initiating antiviral immune responses in dendritic cells [17]. In our study, we found that the PCK2 GpG island was hypomethylated by TET2 elevation, which promoted PCK2 transcription. It has been reported that PCK overexpression is dependent on 5mC, but the specific mechanism has not been elucidated [18]. In contrast, our study demonstrated that PCK2 overexpression was dependent on TET2-induced CPG island hypomethylation.

The involvement of m^6^A in the occurrence of inflammatory responses has also been reported, but the function is not the same; some say it inhibits inflammation [19], and others say it promotes inflammation [20]. The activation of KCs in our study was also accompanied by elevated m^6^A modification. The exercise of m^6^A function is associated with the three types of m^6^A regulators and the target genes that undergo m^6^A changes. The increase in METTL14 and the increase in METTL14 mediate the increase in the m^6^A modification of SOCS1, and the increase in SOCS1 mRNA stability through YTHDF1 is necessary to maintain the negative feedback control of macrophage activation in response to bacterial infection [21]. For example, METTL3 increased the m^6^A modification of TAB3 and promoted the stability of TAB3 in an IGF2BP2-dependent mechanism, leading to the development of nephritis [22]. In the model of KC activation, METTL3/METTL14 was upregulated, mediated the increased m^6^A modification of PCK2 3′UTR, and promoted mRNA stability via IGF2BP1, leading to high PCK2 expression. The high expression of PCK2 may play an accessory role in the inflammatory response. In another study, they found that the m^6^A site of PCK2 3′UTR was recognized by YTHDF2 to promote PCK2 mRNA decay in rabbits [23]. This may be related to the choice of model animals and the treatment of the model. Collectively, we provide support for the post-transcriptional regulation of PCK2.

Both 5mC and m^6^A are involved in the regulation of PCK2, but the relationship between 5mC and m^6^A is unclear. Studies have shown that there is a crosstalk between 5mC and m^6^A [24,25]. Then, there were reports that m^6^A can recruit FXR1, which can bind to TET1, thereby reducing 5mC modification [26]. In our study, we found that 5mC was located upstream of m^6^A. A similar conclusion was obtained in a model of alcohol-induced nephritis, where the 5mC-related enzyme DNMT1 increased the methylation of the FTO GpG island, promoted FTO transcription, and reduced the m^6^A modification of PPAR-α and YTHDF2 induced RNA degradation [27]. In our model, the demethylase TET2 of 5mC mediates the hypomethylation of the CpG islands of METTL3 and METTL14, promoting their transcription and enhancing the m^6^A modification of the PCK2 3′UTR. Although similar results were obtained, the enzymes involved were not the same.

In conclusion, we provide evidence that the mechanism of 5mC promotes PCK2 expression at the transcriptional level and that m^6^A promotes PCK2 expression at the post-transcriptional level during KC activation. We found that 5mC and m^6^A can be used as the key targets for the regulation of PCK2. This study provides a new target for the treatment of liver inflammation. In the future, we will extend the in vitro experiments to in vivo to provide new therapeutic targets for the control of hepatitis.

## 4. Materials and Methods

### 4.1. Cell Culture and Treatment

Mouse KC lines (BeNa Culture Collection, Beijing, China; BNCC340733) were cultured in RPMI 1640 (Wisent, SaintJean-Baptiste, QC, Canada) containing 10% fetal bovine serum (TransGen Biotech, Beijing, China) and 1% penicillin/streptomycin (TransGen Biotech, China) at 37 °C under 5% CO_2_. Cells were cultured to 75% confluence and then treated with 500 ng/mL LPS (L2880, Sigma-Aldrich, St. Louis, MO, USA) for 12 h.

### 4.2. Cytokine Quantification

IL-1β (EK206/3-02) and IL-6 (EK201B/3-48) in cell culture supernatant were quantified using enzyme-linked immunosorbent assay kits purchased from Multisciences (Shanghai, China) according to the manufacturer’s instructions.

### 4.3. RNA Isolation and Real-Time qPCR

Total RNA was isolated from KC line samples by using TRIzol Reagent (TSP401; Beijing Tsingke Biotech Co., Ltd., Beijing, China). One μg of RNA was reverse-transcribed into complementary DNA by using a TransScript Uni All-in-One First-Strand cDNA Synthesis SuperMix for qPCR kit (AU341; TransGen Biotech, China). The complementary DNA (1:20, *v*:*v*) was used for real-time qPCR by using a Mx3000P Real-Time PCR System (Stratagene, San Diego, CA, USA). All primers (Table S1) were synthesized by Tsingke Biotech (China). GAPDH, which was not affected by treatment, was chosen as a reference gene. Data were analyzed by using the method of 2^−ΔΔCT^.

### 4.4. Protein Extraction and Western Blot Assay

KCs were harvested using RIPA lysis buffer (BD0032, Bioworld, Visalia, CA, USA) containing protease inhibitor cocktail (b14001, Selleckchem, Houston, TX, USA) and incubated on ice for 10 min. The protein concentrations were measured by a BCA Protein Assay kit (TransGen Biotech, Beijing, China). Protein samples (30 μg) were used for electrophoresis on a 10% sodium dodecyl sulfate polyacrylamide gel electrophoresis gel and transferred onto a nitrocellulose membrane. The primary antibodies used for Western blot analysis are listed in Table S2. β-actin was selected as an internal control. Images were captured by VersaDoc 4000MP system (Bio-Rad, Hercules, CA, USA), and the band density was analyzed with Quantity One software V4.6.6 (Bio-Rad, Hercules, CA, USA).

### 4.5. DNA 5mC and RNA m^6^A Dot Blot Assay

DNA samples were diluted to 250 ng/μL and heated at 95 °C for 10 min for denaturation. Samples were immediately placed on ice for 5 min, and 1 µL was loaded per dot on a Hybond-N^+^ membrane (GE Healthcare, Chicago, IL, USA). For m^6^A dot blotting, a 500 ng RNA sample was denatured at 95 °C for 5 min and transferred onto a Hybond-N^+^ membrane. The DNA and RNA dot blots were stained with 0.02% methylene blue (in 0.3 mol/L sodium acetate, pH = 5.2) as loading controls. After UV cross-linking, the membranes were washed with TBST buffer, blocked with 5% non-fat milk, and incubated with anti-5mC antibody (AB10805, Abcam, Hangzhou, China, diluted 1:1000) or anti-m^6^A antibody (AB151230, Abcam, Cambridge, MA, USA, diluted 1:1000) overnight at 4 °C. Then, the membrane was incubated with Goat Anti-Mouse IgG (BL001A, Biosharp, Hefei, China, diluted 1:200,000) at room temperature for 2 h. The signals were visualized by a chemiluminescence system (Bio-Rad, USA), and the dot density was analyzed with Quantity One software (Bio-Rad, USA).

### 4.6. Methylated DNA Immunoprecipitation (MeDIP) Assay

Methylated DNA immunoprecipitation (MeDIP) analysis was performed as previously described [28]. Purified genomic DNA from KCs was fragmented to a mean size of 350 base pairs (bp), and 1 μg of fragmented DNA was heat-denatured and immunoprecipitated with a 5-mC antibody (AB10805, Abcam, USA). The precipitated immune complexes were captured by pretreated protein G agarose beads (40 μL, 50% slurry, sc-2003, Santa, CA, USA), and purified MeDIP DNA was used to amplify the proximal promoter sequences of PCK2, METTL3, and METTL14 genes by real-time PCR (Table S3). The CpG islands on the 5′-flanking sequences of the PCK2 (55782477-55787477) gene were predicted with Methprimer http://www.urogene.org/methprimer/ (accessed on 16 April 2024).

### 4.7. SELECT for Site-Specific Detection of m^6^A

SRAMP (http://www.cuilab.cn/sramp accessed on 20 April 2024) was used to predict the potential m^6^A sites on PCK2 mRNA based on the previously published m^6^A-seq databases [11,29]. Three very high/high confidence m^6^A sites were predicted. Among these 3 sites, site 3 was significantly affected by LPS stimulation and was thus selected for site-specific m^6^A quantification by using a single-base elongation and ligation-based qPCR amplification method (termed “SELECT”) [30]. Probes and primers used in the SELECT assay are listed in Table S4.

### 4.8. siRNA Transfection and Inhibitor Treatment

KCs at 60% confluence were transfected with siRNAs specifically designed and verified to knockdown the expression of TET2 (5′-CCCACAAGGACCAACAUAATT-3′), IGF2BP1 (5′-TATTCCACCCCAGCTCCGAT-3′) and YTHDF2 (5′-GCAAACTTGCAGTTTATGTAT-3′) by Beijing Tsingke Biotech Co., Ltd. (China). Cells were seeded onto a 6-well plate for transfection using Lipofectamine 2000 (Invitrogen, Waltham, MA, USA), following the manufacturer’s instructions.

### 4.9. RNA Decay Assay

KCs were cultured in 6-well plates followed by the treatment with or without LPS or m^6^A inhibitor cycloleucine (52-52-8, Sigma-Aldrich, USA). The de novo transcription was inhibited by actinomycin D (HY-17559, MCE, Junction, NJ, USA) at a final concentration of 5 μg/mL, and cells were collected for RNA extraction at different time points after actinomycin D treatment. The isolated total RNA was used for RT-qPCR to quantify the relative abundance of PCK2 mRNA at 3 and 6 h relative to 0 h.

### 4.10. Dual-Luciferase Reporter Assay

A pRL-TK vector was used to construct PCK2-3′UTR-WT and PCK2-3′UTR-Mut plasmids by Beijing Tsingke Biotech Co., Ltd. (China). The m^6^A site in PCK2 3′UTR that was significantly hypermethylated in LPS-activated KCs was mutated from A to T. KCs were transfected with respective plasmids for 12 h and treated with or without LPS for another 12 h. Cells were subjected to luciferase activity analyses following the instructions of the dual luciferase reporter assay kit (DL101-1, Vazyme, Nanjing, China).

### 4.11. Statistical Analysis

All data are presented as mean ± SE. All experiments were repeated at least twice. The differences between groups were analyzed using Student’s *t*-test or 2-way analysis of variance followed by Tukey’s test for multiple comparisons with SPSS 20.0 (IBM, New York, NY, USA). The differences were considered statistically significant when *p* < 0.05.

## Data Availability

Data is contained within the article and Supplementary Materials.

## Supplementary Materials

The supporting information can be downloaded at https://www.mdpi.com/article/10.3390/ijms25189894/s1.
