# Supplementary material for: DNA 5mC and RNA m6A Collaborate to Upregulate Phosphoenolpyruvate Carboxykinase 2 for Kupffer Cell Activation
Source: Int J Mol Sci. 2024 Sep 13;25(18):9894. doi: 10.3390/ijms25189894 (PMC11432282; doi:10.3390/ijms25189894)
Supplement: Supplementary file 1 [file ijms-25-09894-s001.zip › ijms-3143075-supplementary.pdf]

Figure S1

Supplemental Figure S1. A Verification of TET2 knockdown effect B Knockdown of YTHDF2 failed to restore LPS-induced increase in PCK2 (n = 3). Values are means ± SE, ** $p < 0.01$. C METTL3, METTL14, FTO, YTHDF1, YTHDF2, YTHDF3 and IGF2BP1 protein expression after TET2 knockdown (n = 3). D DNMT1, DNMT3A and TET2 protein expression after treatment with CYC (n = 3). Values are means ± SE, Values are means ± SE, * $p < 0.05$ and ** $p < 0.01$.

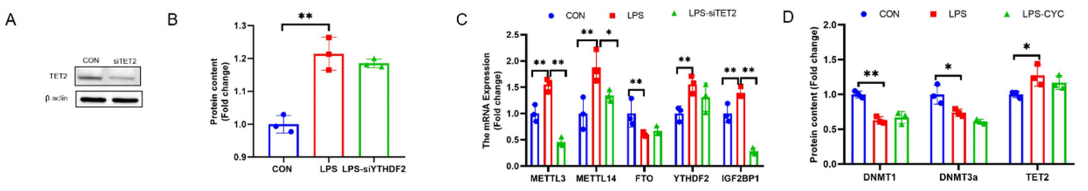

Table S1. Nucleotide sequences of primers

Target	Primer sequences (5'to 3')		Used for
IL-1β	F: AATGCCACCTTTTGACAGTGATG	R: GGAAGGTCCACGGGAAAGAC	Real-time PCR
IL-6	F: GGAAGGTCCACGGGAAAGAC	R: GGAAGGTCCACGGGAAAGAC	Real-time PCR
HK2	F: TGATCGCCTGCTTATTCACGG	R: AACCGCCTAGAAATCTCCAGA	Real-time PCR
PFKP	F: GCCGTGAAACTCCGAGGAA	R: GTTGCTCTTGACAATCTTCTCATCAG	Real-time PCR
PKM2	F: CGCCTGGACATTGACTCTG	R: GAAATTCAGCCGAGCCACATT	Real-time PCR
PCK1	F: AGCATTCAACGCCAGGTTC	R: CGAGTCTGTCAGTTCAATACCAA	Real-time PCR
PCK2	F: TGCCGTAGCATCCAAACCCT	R: CTGCGCGCCACAAAGTCT	Real-time PCR
DNMT1	F: CGGACAGTGACACCCTTTCA	R: TCCTGGTCTCTCTCCTCTGC	Real-time PCR
DNMT3a	F: GAGAACCGTGGCAAGGAAGA	R: GGTTCACTCCGCTTCTCCAA	Real-time PCR
DNMT3b	F: GATGAGGAGAGCCGAGAACG	R: CACCTCCAGACACTCCACAC	Real-time PCR
TET1	F: AACATGCACAACGGAAGCAC	R: GTGTTGTGTGAACCTGATTTATTGT	Real-time PCR
TET2	F: CCCAAAGCTACTCGCATCAGA	R: CCCTGACAACAACAGTTTCTAGTT	Real-time PCR
TET3	F: CCGTGACTGTGCTCTCAACT	R: TCCTCCATGAGTTCCCGGAT	Real-time PCR
METTL3	F: GCTCCATCCAGGCCCATAAG	R: CCCACTCACCGTATCGATGG	Real-time PCR
METTL14	F: GTGATTCTCCTGGAGCCACC	R: TGGGGTCCAGAGTCTTCGTT	Real-time PCR
FTO	F: TGAAGGTAGCGTGGGACATAGA	R: TGAAGGTAGCGTGGGACATAGA	Real-time PCR
YTHDF1	F: ACAAGCGTTGACCCTCAGAGA	R: TGTTCCCCAAGCTGAGAAGG	Real-time PCR
YTHDF2	F: TCCTACTCTCTGGGTGAGGC	R: GCGTAATTGCTGCTGTAGCC	Real-time PCR
YTHDF3	F: CCACCAACACTGGTGCAAAG	R: GCCCACACCCCTATTACGAG	Real-time PCR
IGF2BP1	CCCGCAGACTTGGAGAAAGT	R: CACTTCCCATCGGAGCTGAG	Real-time PCR
GAPDH	F: TCTCCTGCGACTTCAACA	R: TGTAGCCGTATTCATTGTCA	Real-time PCR

Table S2. The Primary Antibodies Used for Western Blot Analysis

Primary Antibody	Company	Catalogue NO.	Dilution
METTL3	Abcam	AB98009	1:3000
METTL14	Abcam	AB98116	1:1000
FTO	Abcam	AB77547	1:1000
p-p65	CST	3033	1:1000
YTHDF1	Proteintech	17479-1-AP	1:2000

YTHDF2	Proteintech	24744-1-AP	1:2000
YTHDF3	Proteintech	25537-1-AP	1:1000
DNMT1	Proteintech	24206-1-AP	1:1000
DNMT3a	Abclonal	A11791	1:1000
TET2	Proteintech	21207-1-AP	1:1000
PCK1	Proteintech	16754-1-AP	1:1000
PCK2	Abcam	ab70359	1:1000
β-actin	Bioworld	AP0060	1:10000

Table S3. Nucleotide sequences of primers

Target	Primer sequences (5'to 3')		Used for
PCK2	F: GCATTCGACATCTCTTGGTTGC	R: TTCAATTTGGGATCCGCCCA	MeRIP-PCR
METTL3	F: TTTCACCGGCATCGTGAGAA	R: GGTGGCACAGACATTCCGTA	MeRIP-PCR
METTL14	F: TGTTTCCTGGTTTGGCAGGT	R: CACTAATGCTCCCTCCCACCC	MeRIP-PCR

Table S4. Nucleotide Sequences of SELECT Method

Target	Sequences (5'to 3')	
PCK2 3'UTR Motif 1 X site	Up probe:<br>tagccagtaccgtagtgcgtgTTTTAATGTCAGATTGTTCATG	Down probe:<br>TTTCTGTGAGCCTGGGGGcagaggctgagtcgctgcat
PCK2 3'UTR Motif 2 X site	Up probe:<br>tagccagtaccgtagtgcgtgCACACATTTTAATGTCAGATTG	Down probe:<br>TCATGTTTTCTGTGAGCCTGGcagaggctgagtcgctgcat
PCK2 3'UTR Motif 3 X site	Up probe:<br>tagccagtaccgtagtgcgtgCCAACACTCACACATTTTAATG	Down probe:<br>CAGATTGTTCATGTTTTCTGTGcagaggctgagtcgctgcat
PCK2 3'UTR Motif N site	Up probe:<br>tagccagtaccgtagtgcgtgTGTCAGATTGTTCATGTTTTCT	Down probe:<br>TGAGCCTGGGGGTTCTCTcagaggctgagtcgctgcat
qPCR	Forward Primer:<br>ATGCAGCGACTCAGCCTCTG	Reverse Primer:<br>TAGCCAGTACCGTAGTGCGTG
